# Indian Plant Germplasm on the Global Platter: An Analysis

**DOI:** 10.1371/journal.pone.0126634

**Published:** 2015-05-14

**Authors:** Sherry R. Jacob, Vandana Tyagi, Anuradha Agrawal, Shyamal K. Chakrabarty, Rishi K. Tyagi

**Affiliations:** 1 Division of Germplasm Conservation, ICAR-National Bureau of Plant Genetic Resources, New Delhi, India, 110012; 2 Germplasm Exchange Unit, ICAR-National Bureau of Plant Genetic Resources, New Delhi, India, 110012; 3 Tissue Culture and Cryopreservation Unit, ICAR-National Bureau of Plant Genetic Resources, New Delhi, India, 110012; 4 ICAR-National Bureau of Plant Genetic Resources, Regional Station, Hyderabad, 500030, India; Julius Kuehn-Institute (JKI), GERMANY

## Abstract

Food security is a global concern amongst scientists, researchers and policy makers. No country is self-sufficient to address food security issues independently as almost all countries are inter-dependent for availability of plant genetic resources (PGR) in their national crop improvement programmes. Consultative Group of International Agricultural Research (CGIAR; in short CG) centres play an important role in conserving and distributing PGR through their genebanks. CG genebanks assembled the germplasm through collecting missions and acquisition the same from national genebanks of other countries. Using the Genesys Global Portal on Plant Genetic Resources, the World Information and Early Warning System (WIEWS) on Plant Genetic Resources for Food and Agriculture and other relevant databases, we analysed the conservation status of Indian-origin PGR accessions (both cultivated and wild forms possessed by India) in CG genebanks and other national genebanks, including the United States Department of Agriculture (USDA) genebanks, which can be considered as an indicator of Indian contribution to the global germplasm collection. A total of 28,027,770 accessions are being conserved world-wide by 446 organizations represented in Genesys; of these, 3.78% (100,607) are Indian-origin accessions. Similarly, 62,920 Indian-origin accessions (8.73%) have been conserved in CG genebanks which are accessible to the global research community for utilization in their respective crop improvement programmes. A total of 60 genebanks including 11 CG genebanks have deposited 824,625 accessions of PGR in the Svalbard Global Seed Vault (SGSV) as safety duplicates; the average number of accessions deposited by each genebank is 13,744, and amongst them there are 66,339 Indian-origin accessions. In principle, India has contributed 4.85 times the number of germplasm accessions to SGSV, in comparison to the mean value (13,744) of any individual genebank including CG genebanks. More importantly, about 50% of the Indian-origin accessions deposited in SGSV are traditional varieties or landraces with defined traits which form the backbone of any crop gene pool. This paper is also attempting to correlate the global data on Indian-origin germplasm with the national germplasm export profile. The analysis from this paper is discussed with the perspective of possible implications in the access and benefit sharing regime of both the International Treaty on Plant Genetic Resources for Food and Agriculture and the newly enforced Nagoya Protocol under the Convention on Biological Diversity.

## Introduction

Plant genetic resources (PGR) comprise any plant material of actual or potential value, containing functional units of heredity [1]. According to an estimate, out of 250,000 higher plant species described taxonomically so far [2], about 115,000 are PGR (46%) and 35,000 (14%) species are cultivated [3]. Plant Genetic Resources for Food and Agriculture (PGRFA) (also known as crop diversity) refer to the plants utilized for food and agriculture, which are astonishingly few; humanity depends on less than a dozen flowering plants for 80% of its caloric intake [4]. Recent reports have shown that loss of biodiversity through human activities has been faster over the past 50 years than ever before in human history and more alarmingly, up to 75% of the genetic diversity of crops has already disappeared [5]. In the past century or so, stakeholders such as breeders, researchers and farmers have consciously gathered and saved the seeds of hundreds of landraces, primitive varieties, modern varieties and wild relatives of crop species. These are stored *ex situ* in national and international genebanks worldwide [5] and form the primary source for advances in plant breeding, generating billions of dollars in profits. For example, cross breeding a single wild species of rice, *Oryza nivara* originating from the fields of northern plains of India, which was found after screening of more than 6,000 seed bank accessions, has provided protection against grassy stunt virus disease in almost all tropical rice varieties in Asia in recent decades [6]. Such and several other gene-rich PGR exist in agro-biodiversity hotspots, including India.

India is a floristically and genetically rich nation. It is endowed with an immense richness of PGR because of its varied geography, diverse ecosystems and a rich agricultural heritage linked with its ethnic diversity. India has about 46,042 species of flowering and non-flowering plants (more than 11% of the world’s known flora), making it rank tenth in the world and fourth in Asia in terms of plant diversity [7]. Flowering plants constitute 17,527 species (7% of the world total). The Indian gene centre is one of the 12 mega diversity centres of the world [8] and it has Eastern Himalayas, Western Ghats, Indo-Burma and Sundaland (Nicobar Islands) regions as four ‘biodiversity hotspots’ out of 34 spread around the world [9]. Besides the native ethnic PGR diversity, India has been richly complemented by exotic introductions made since centuries. Over the period, these introduced genetic resources have been subjected to natural selection and adaptation, which led to the evolution of one of the most heterogeneous genetic pools within a political boundary, with all the diversity being cohesively distributed into 15 prevalent agro-climatic zones in India [10]. Each agro-climatic zone is an ecological niche dominated by species that are specific to the cropping system that has evolved in a particular region. India also has a rich history of PGR exchange activities which dates back to the early 20th century. However, they were scientifically and formally executed with the creation of Division of Plant Introduction at Indian Agricultural Research Institute (IARI), New Delhi, in the 1960s under the aegis of the Indian Council of Agricultural Research (ICAR). This was subsequently upgraded to a full-fledged institute, the ICAR-National Bureau of Plant Genetic Resources (ICAR-NBPGR) in 1976. Importantly, it houses the National Genebank (NGB), established during 1985–86 for *ex situ* conservation [11].

Globally, the Consultative Group on International Agricultural Research (CGIAR; in short CG) centres established 11 genebanks for conservation and use of PGR which are now held ‘in-trust’ for the world community under signed agreements with FAO in 1994. In addition to this, about 1,750 individual genebanks are reported worldwide; out of which 130 genebanks hold more than 10,000 accessions and 8 have more than 100,000 accessions [5]. The Svalbard Global Seed Vault (SGSV) was established in 2008 to provide the safety net for the international conservation system of PGR, managed in partnership by the Government of Norway, the Nordic Genetic Resources Centre (NordGen) and Global Crop Diversity Trust (GCDT). According to FAO [5] the four largest genebanks at national level (in decreasing order of holdings) are—(i) National Centre for Genetic Resources Preservation (NCGRP) in United States of America, (ii) Institute of Crop Germplasm Resources, Chinese Academy of Agricultural Sciences (ICGR-CAAS) in China, (iii) ICAR-NBPGR in India and (iv) N.I. Vavilov All Russian Scientific Research Institute of Plant Industry (VIR) in the Russian Federation. However, currently the ICAR-NBPGR genebank holds 392,163 germplasm accessions [12] belonging to 1761 species (excluding trial materials and safety duplicates deposited by CG genebanks) and thus becomes the second largest, with ICGR-CAAS genebanks having 374,627 accessions belonging to 898 species [13] moving to the third position.

The accumulation of germplasm in genebanks, introduction of intellectual property rights (IPR) with respect to biological resources, the imbalance in availability of technology with the developed nations and the genetic resources with developing nations (North-South divide) prompted a profound change in the legal landscape of biodiversity, in general, and PGR in particular, with regard to ownership, access and benefit sharing (ABS) [14]. Three global international agreements that directly impact the access, exchange, conservation and utilization of PGR today are the Convention on Biological Diversity (CBD), 1993; the International Treaty on Plant Genetic Resources for Food and Agriculture (ITPGRFA), 2004 and the Nagoya Protocol on Access to Genetic Resources and the Fair and Equitable Sharing of Benefits arising from their Utilization (NP), 2014. The ITPGRFA is the legal instrument for ABS with regard to PGRFA belonging to 64 crops mentioned in Annex I of the Treaty. The NP aims at providing fair and equitable share of benefits arising from the utilization of all genetic resources. It also makes it mandatory to grant the share of benefits to local people during commercial utilization of any genetic material. These policy developments have, or rather have been discussed to have, an apparent effect on the germplasm exchange patterns of member countries which, otherwise, had been major donors for many international *ex situ* collections that are globally utilized in various crop breeding programmes. Whether or not ITPGRFA facilitated access to PGR is also still a mooted question [15].

India has ratified all the three treaties (CBD, ITPGRFA and NP) and also enacted its own Biological Diversity Act (BDA), in 2002 that governs the ABS mechanisms of genetic resources held within its political boundaries. The BDA functions as the nodal body for granting approval for use of Indian biological resources by researchers of other countries. It delineates the conditions under which persons, commercial firms and other institutions can access biological resources occurring in India and the knowledge associated with the biological resource, for research or for commercial utilization, or for bio-survey and bio-utilization [16]. A categorical analysis on conservation and the utilization of Indian-origin PGR in international breeding programmes has not been made till date due to the absence of a stringent feedback mechanism or authenticated data about the use of germplasm by international breeders/researchers. However, Indian-origin germplasm of crops and their wild relatives possessed and provided by India and conserved in the CG genebanks and national genebanks of other countries, can be considered as a true indicator of India’s contribution to the global genetic pool and an apparent indicator of its utilization in international breeding programmes, owing to their mostly unrestricted access by the global agricultural research community. Therefore, we collected, collated and analysed information about the Indian-origin PGR available in national and international genebanks which are accessible to researchers globally for conservation and utilization in crop improvement programmes. The trigger for the present study was multi-fold: (i) it aims at assessing whether changes in the recent legal and administrative policies *vis-a-vis* PGR have, in any way, hindered global use of Indian germplasm, (ii) *prima facie* limitations for access to Indian germplasm are often reported [17, 18] but in fact it is a matter of debate and merits an unbiased analysis, (iii) how much plant germplasm has been exported by India and how many countries are the beneficiaries thereof, and (iv) proposing some mechanisms to enhance PGR utilization with pragmatic ABS approaches, in the light of both, the ITPGRFA and the NP.

## Methodology

### Analysis of germplasm accessions conserved in global genebanks

A concise assessment of these data has been carried out using data accessed through the Genesys Global Portal on Plant Genetic Resources [19]. The database values mentioned in the paper correspond to the information provided by the website as on August 31, 2014. All the analyses were conducted online. The ‘accession browser’ option on the website’s homepage was used as the basic data source. The total list of Indian-origin accessions conserved in the enlisted genebanks was derived using the ‘add filter’ option and by designating the ‘country of origin’ as ‘IND, India’. The ‘overview’ option in the ‘accession browser’ page provided the compiled percentage of Indian-origin accessions in the total germplasm collection. Similarly, the ‘statistical overview’ of the Indian-origin accessions was used for identifying the ‘major holding institutes’ of Indian-origin germplasm. This webpage mentions the total number and percentage of Indian-origin accessions held by 11 individual institutes (four CG genebanks and eight national genebanks) and the rest of the ‘holding institutes’ were categorised in the ‘others’ sub-heading. For the present analysis, instead of restricting to these four CG genebanks, we included all 11 CG genebanks mentioned by the website, so as to avoid missing any relevant information. Information on each CG genebank was individually accessed through their respective hyperlinks on the mentioned webpage. Indian-origin accessions conserved by these CG genebanks were obtained using the ‘add filter’ option as mentioned above.

In case of major national genebanks that hold Indian-origin germplasm, the information from the ‘overview’ table was compared with the database of WIEWS (World Information and Early Warning System on Plant Genetic Resources for Food and Agriculture). As per WIEWS database, the major institutes that hold Indian-origin germplasm were the genebanks belonging to the United States of America (USA), Russia, Taiwan, Germany, United Kingdom (2), Poland, and the Czech Republic. Hence, we have considered these eight sources for the present analysis on Indian-origin germplasm conserved in national genebanks. Within USA, a total of 36 genebanks have been listed, out of which 26 genebanks belonging to the United States Department of Agriculture—Agricultural Research Service, are the major holders of Indian germplasm. The information of germplasm of the above 26 genebanks was pooled and has been presented in this document as ‘USDA genebanks’.

### Analysis of germplasm accessions deposited and conserved in the Svalbard Global Seed Vault

The data on germplasm deposited by the major national genebanks at the Svalbard Global Seed Vault (SGSV), Norway, which is the nodal point for safety duplication of the world’s unique crop genetic resources, were accessed from its official website [20]. The link for ‘depositor institutes’ provided on the homepage of the website was accessed and data of each institute was filtered based on ‘country name’, to obtain the data on accessions of Indian-origin. Out of the total 60 depositor organizations mentioned on the website, 24 institutes were found to have submitted Indian-origin accessions, the details of which were compiled for this analysis. However, the summary table of Indian-origin accessions which can be accessed through the ‘country of origin’ link provided on the homepage, mentions 31 genebanks as donors of Indian-origin germplasm, which did not match with our derived data.

### Analysis of germplasm accessions exported by India

In order to have a direct assessment of the impact of various policy regimes on India’s germplasm supply pattern, we attempted to analyse the data on year-wise export of germplasm documented by ICAR-NBPGR from 1976 to 2013. These data have been further validated by an analysis of the actual number of germplasm requests received and processed by ICAR-NBPGR, which was the sole authority for processing Indian germplasm for export, till 2004. From 2005 onwards, ICAR-NBPGR has been dealing with only those export indents that come under the purview of collaborative research programmes and the National Biodiversity Authority (NBA) established under the aegis of BDA handles all other requests. For the present analysis, we have used only the data on requests/indents processed by ICAR-NBPGR, which, from 2005 onwards, includes only those indents placed under collaborative research programmes.

The details of germplasm exported by India were compiled from the individual Annual Reports of ICAR-NBPGR (available as authentic records only from 1976 onwards, which is the year of inception of NBPGR) [21]. The export requests received and processed at NBPGR were also compiled from authenticated official records.

## Results and Discussion

### Indian-origin plant germplasm in CG genebanks and other national genebanks

**Fig 1:**
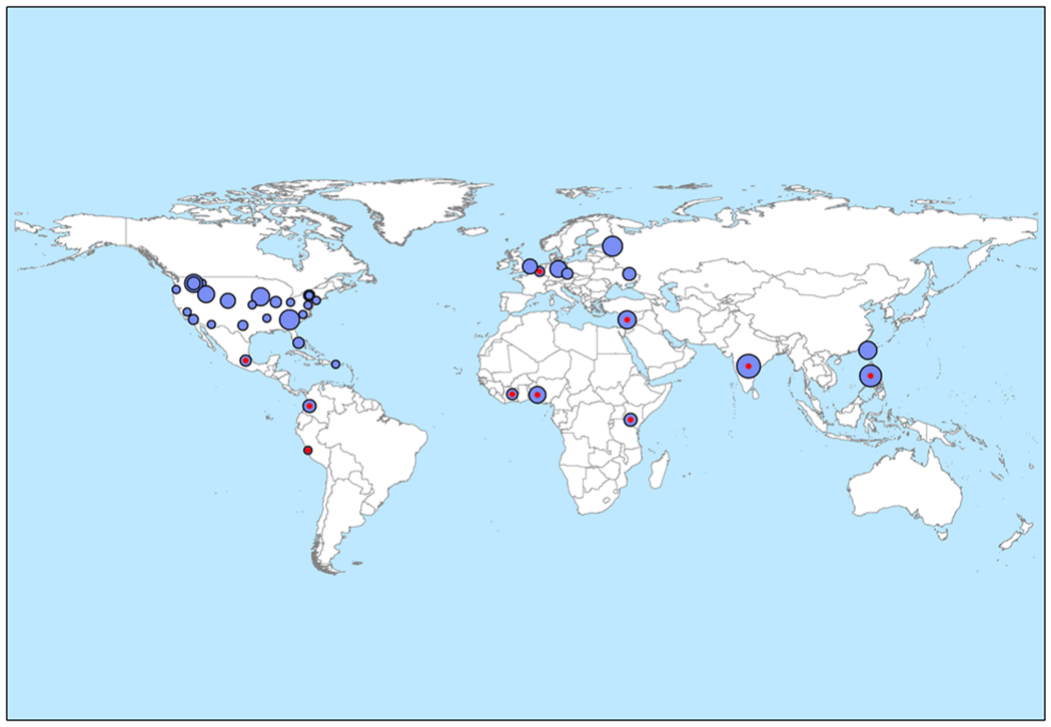
Global distribution of Indian-origin germplasm conserved by national and international genebanks. The radius of each circle is relative to the number of Indian-origin accessions conserved, and the circle sizes were partitioned into 10 size classes. Circles with red indicator represent the CG genebanks.

Data sourced from the Genesys portal revealed the conservation status of 2,802,770 germplasm accessions held in genebanks belonging to 446 organizations. Out of these, the Indian-origin germplasm amounts to 106,007 accessions (3.78% of total reported germplasm), of which 62,920 accessions are held in CG genebanks. A detailed source-wise estimate of accessions held by 11 CG genebanks revealed that 8.73% of their total conserved germplasm (720,699) are of Indian-origin (Table 1). A study based on CGIAR’s System-Wide Information Network for Genetic Resources database (SINGER, now included in Genesys) and other PGR databases had shown that India contributed the highest number of PGR accessions to CG genebanks (66,864; 9.28%) and ranks first in the list of contributors [22]. The other major holders of Indian-origin germplasm are the genebanks in USA, Russia, Taiwan, Germany, UK, Poland and the Czech Republic (Table 2). Amongst these eight major genebanks, the AVRDC genebank of Taiwan has the highest (7.77%) proportion of Indian-origin germplasm, followed by John Innes Centre, UK (6.43%) and USDA genebanks (3.61%). The geographical distribution of the total globally conserved Indian-origin germplasm, as reported by Genesys, has been mapped and depicted in Fig 1.

**Table 1:** Germplasm of Indian-origin conserved in CGIAR genebanks.

S. No.	CGIAR Genebank	Total no. of accessions	Accessions of Indian origin	
			No.	%
1	International Crop Research Institute for the Semi-Arid Tropics, India	119,524	37,470	35.54
2	International Rice Research Institute, The Philippines	131,862	17,824	16.81
3	International Centre for Agricultural Research in Dry Areas, Syria	147,118	3,747	3.53
4	International Institute of Tropical Agriculture, Nigeria	27,232	2,276	8.36
5	International Livestock Research Institute, Ethiopia	20,229	501	2.48
6	Centro Internacional de Agricultura Tropical, Colombia	64,721	422	0.65
7	Centro Internacional de Mejoramiento de Maíz y Trigo, Mexico	164,320	318	0.19
8	West African Rice Development Association, Ivory Coast	26,098	299	1.15
9	*Musa* International Transit Centre, Bioversity International, Belgium	1,529	54	3.53
10	Centro Internacional de la Papa, Peru	16,061	9	0.06
11	Information and Communication Division, International Center for Research in Agroforestry, Kenya	2,005	0	0
	**Total**	**720,699**	**62,920**	**8.73**

The table lists the number of accessions of Indian-origin, conserved in the 11 CG genebanks. The genebanks are listed by ‘No. of accessions of Indian origin’ in descending order.

**Table 2:** Germplasm of Indian-origin conserved in major national genebanks.

S. No.	International Genebank	Total no. of accessions	Accessions of Indian-origin	
			No.	%
1	USDA Genebanks, USA	625,112	22,582	3.61
2	N.I. Vavilov All-Russian Scientific Research Institute of Plant Industry, Russia	346,415	8,145	2.35
3	Asian Vegetable Research and Development Center, Taiwan	60,883	4,729	7.77
4	Leibniz Institute of Plant Genetics and Crop Plant Research, Germany	137,010	2,233	1.63
5	Department of Applied Genetics, John Innes Centre, Norwich Research Park, UK	26,669	1,714	6.43
6	Plant Breeding and Acclimatization Institute, Poland	67,980	428	0.62
7	Millennium Seed Bank Project, Seed Conservation Department, Royal Botanic Garden, Kew, UK	46,689	335	0.72
8	Division of Genetics and Plant Breeding, Research Institute of Crop Production, Czech Republic	43,151	274	0.63

The table lists the number of accessions of Indian origin, conserved in the major national genebanks. The genebanks are listed by ‘No. of accessions of Indian origin’ in descending order.

### Indian-origin plant germplasm deposited and conserved in SGSV as safety duplicates

A parallel analysis of the SGSV database revealed a proportionate presence of Indian-origin germplasm in the form of safety duplicate collections. Data drawn from the website shows that SGSV has received 824,625 germplasm accessions from 60 genebanks, amongst which 66,739 germplasm accessions (8.09%), deposited by 24 genebanks, are of Indian origin (S1 Table). The major depositors of Indian-origin germplasm (in decreasing order) are the International Crop Research Institute for the Semi-Arid Tropics (ICRISAT), India; the International Rice Research Institute (IRRI), the Philippines; the National Plant Germplasm System, USA (USDA Genebanks), The World Vegetable Center (AVRDC), Taiwan and the International Centre for Agricultural Research in Dry Areas (ICARDA), Syria. It has been reported that there is a big gap in deposition of germplasm in SGSV by India [18]. Though, India has directly submitted only 25 accessions to SGSV, it should be noted that 66,739 accessions of Indian-origin are currently being held in SGSV, which have been deposited through CG genebanks and other national genebanks. IRRI has deposited its almost entire rice germplasm collection (116,668) in SGSV, wherein 16,220 accessions belonging to 18 species (including crosses) have their origin in India. Similarly, 33,424 accessions out of 104,000 accessions of pearl millet, sorghum, pigeonpea, chickpea, groundnut and small millets crops deposited by ICRISAT, are of Indian-origin. Considering the total accessions (824,625) deposited in SGSV by 60 genebanks (CG genebanks and national genebanks), the theoretical mean value of germplasm accessions contributed by an individual genebank may be calculated as 13,744 accessions. Hence, in principle, Indian-origin accessions have been deposited 4.85 times in comparison to the mean value of accessions deposited by any genebank, including CG genebanks.

The global Indian-origin germplasm collection, as reported in the Genesys portal and SGSV databases, has major representation from crops like *Oryza sativa* L., *Oryza nivara* S.D. Sharma & Shastry, *Cicer arietinum* L., *Cajanus cajan* (L.) Millsp., *Sorghum bicolor* (L.) Moench, *Triticum aestivum* L., *Hordeum vulgare* L., *Pennisetum glaucum* (L.) R.Br., *Setaria italica* (L.) P.Beauv., *Eleusine coracana* Gaertn., *Panicum miliaceum* L., *Echinochloa frumentacea* Link., *Solanum melongena* L., *Cucumis melo* L., *Vigna radiata* (L.) Wilczek, *Pisum sativum* L., *Lens culinaris* Medik., *Linum usitatissimum* L., *Phaseolus vulgaris* L., *Arachis hypogaea* L. and *Amaranthus hypochondriacus* L. Tables 3 and 4 present the details of representation of Indian-origin germplasm of 16 major food crops as depicted in the Genesys and SGSV databases, respectively. It is evident from this data that a large proportion of Indian origin germplasm of pigeonpea (66–70%), foxtail millet (43–58%), pearl millet (28–31%) and finger millet (23–36%) are conserved in global genebanks. Notably, out of these 16 crops, 10 crops belong to Annex 1 of ITPGRFA and for nine crops (rice, sorghum, mungbean, chickpea, eggplant, pearl millet, foxtail millet, pigeonpea, finger millet), India is either the Vavilovian centre of origin or a primary or secondary centre of diversity. Further, according to Genesys, around 50% of the Indian-origin germplasm accessions are traditional cultivars or landraces with defined traits, which form the back-bone of any crop gene pool. Hence, these Indian-origin accessions unequivocally represent the elite components of their respective global genetic diversity, which are directly available to the global community through the CG centres (except for a nominal 3.8% of the total Indian-origin germplasm, which has been marked in the database as ‘*not available for distribution*’) or might have been evolved into structured genotypes that are currently feeding the world population across all the continents. The utilization, to whatever extent, is of course, the result of superior breeding interventions made by multiple stakeholders during the course of the germplasm flow. However, India started receiving its due acknowledgement only with the implementation of the CBD regime.

**Table 3:** Crop-wise details of accessions listed in Genesys portal.

S. No.	Crop	Genesys			CG genebanks		
		Total accessions	Indian origin accessions		Total accessions	Indian origin accessions	
			No.	%		No.	%
1	Pigeonpea	13,773	9,216	66.91	13,588	9,155	67.38
2	*Setaria* sp.	3,246	1,393	42.91	1,694	985	58.15
3	Finger millet	8,154	2,979	36.53	5,969	1,372	22.99
4	Pearl millet	22,866	6,509	28.47	21,477	6,468	30.12
5	Amaranth	6,299	1,387	22.02	51	0	0
6	Chickpea	56,714	10,796	19.04	35,050	8,133	23.2
7	Eggplant	5,474	1,019	18.62	0	0	0
8	*Vigna* sp.	54,690	8,991	16.44	20,452	2,328	11.38
9	Groundnut	28,912	4,550	15.74	15,625	3,754	24.03
10	Lentil	26,653	2,725	10.22	12,463	2,136	17.14
11	Rice	223,530	19,699	8.81	157,959	18,123	11.47
12	Sorghum	99,859	8,620	8.63	37,978	6,228	16.4
13	Sesame	3,286	279	8.49	1	0	0
14	Wheat	388,923	6,048	1.56	140,994	720	0.51
15	Cabbage	14,582	84	0.58	6	0	0
16	Maize	137,350	293	0.21	28,089	4	0.01
	**Total**	**1,094,311**	**84,588**	**7.73**	**491,396**	**59,406**	**12.09**

The table lists the details of 16 crops having the maximum representation in the Genesys portal. The crops are arranged in the decreasing order of percentage of Indian origin accessions in the total accessions list of Genesys Portal. Indian-origin accessions have been also derived separately for total accessions conserved by CG genebanks, from the same portal.

**Table 4:** Crop-wise details of accessions listed in SGSV database.

S. No.	Crop	Total accessions	Indian origin accessions (no.)	Indian origin accessions (%)
1	Pigeonpea	9,948	6,971	70.07
2	*Setaria* sp.	2,602	1,345	51.69
3	Amaranth	1,315	421	32.02
4	Pearl millet	20,983	6,475	30.86
5	Finger millet	7,545	1,974	26.16
6	Chickpea	28,722	7,357	25.61
7	Groundnut	15,823	3,650	23.07
8	Eggplant	2, 224	495	22.26
9	*Vigna* sp.	29,236	5,764	19.72
10	Lentil	12,006	2,121	17.67
11	Sorghum	44,031	5,973	13.57
12	Rice	145,672	16,774	11.51
13	Wheat	154,131	1,106	0.72
14	Sesame	1,733	9	0.52
15	Cabbage	2,038	4	0.2
16	Maize	35,219	17	0.05
	**Total**	**513,228**	**60,456**	**11.78**

The table lists the conservation status of 16 crops mentioned in Table 3, in SGSV. The crops are arranged in the decreasing order of percentage of Indian—origin accessions.

All these Indian-origin germplasm accessions deposited by CG genebanks in SGSV are also available for the global agricultural research community from the respective CG centres. In the case of germplasm sourced from individual countries during the pre-CBD regime, and conserved in CG genebanks, they have been placed under the designation of ‘in-trust’ germplasm, as per the FAO-CGIAR Trust agreement of 1994 whereas the fate of PGR collected and taken away prior to CBD from their country of origin to non-CGIAR genebanks, still remains unresolved. All accessions under the ‘in-trust’ category are available in the public domain with unrestricted access. An analysis of the global distribution of germplasm by the CG centres, as per SINGER database, reveals that the most frequently distributed germplasm (to 119 countries) is the Indian-origin germplasm [22]. The free access has been further corroborated with the implementation of ITPGRFA under which a list of 35 food crops and 29 forage crops (designated as Annex 1 crops) has been mandatorily placed under a multilateral system (MLS), wherein contracting parties (127 members by 2014) have agreed to virtually pool a subset of the genetic resources of the 64 crops and forages to be used for utilization and conservation for research, breeding and training for food and agriculture. India signed and ratified the ITPGRFA in June 2002 and ICAR-NBPGR is serving as the single-window system for exchange of germplasm under the ITPGRFA. The *quid pro quo* system provides facilitated access to the pooled PGRFA of all the other member countries in exchange for putting PGRFA of the listed genera in the system, with minimal transaction costs. Currently in India, the PGR are exchanged as part of projects that are approved by the central government (under Section 5 of BDA). This exemption has been used in cases to approve the transfer of a relatively small number of samples of PGRFA out of India using the Standard Material Transfer Agreement (SMTA) [23]. An inter-ministerial Joint Working Group along with a National Advisory Board for the Management of Genetic Resources are actively working for implementation of the provisions of the MLS in India [24] by taking the measures to include approval of the list of accessions under the MLS from the Government of India as well as the modalities for rapid germplasm exchange for 64 PGRFA listed in the MLS.

### Germplasm exported by India

**Fig 2:**
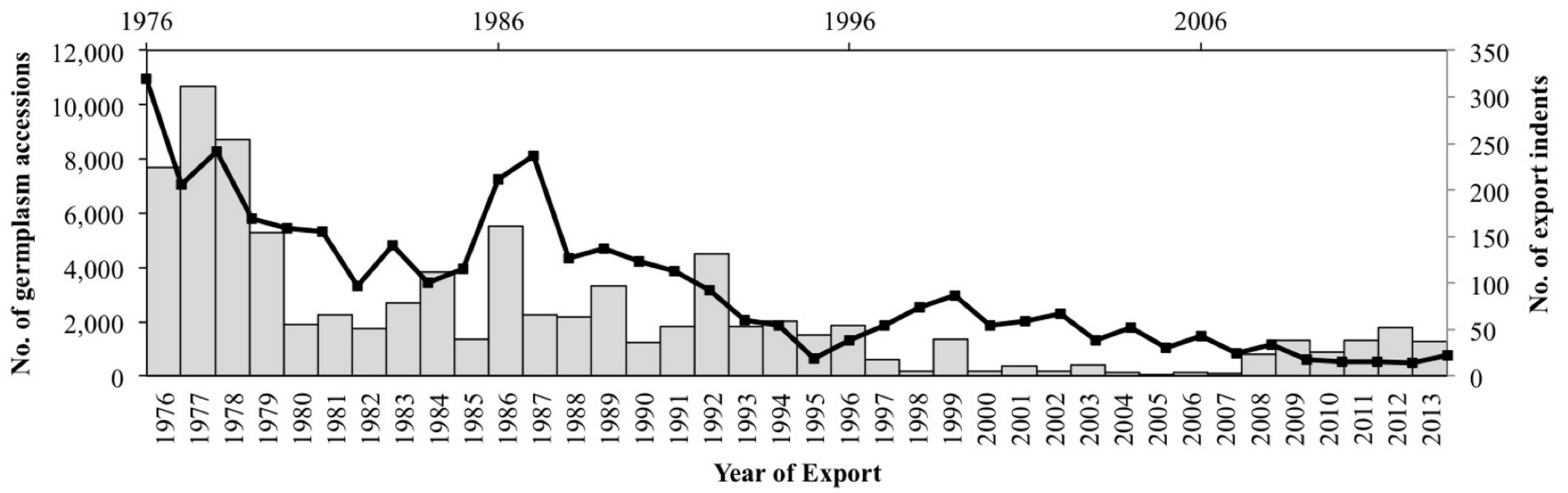
Year-wise export profile of Indian-origin germplasm from India to other countries. The bar graph data represents a total of 91, 403 accessions exported by India. The superimposed line graph represents the number of export indents received by India.

**Fig 3:**
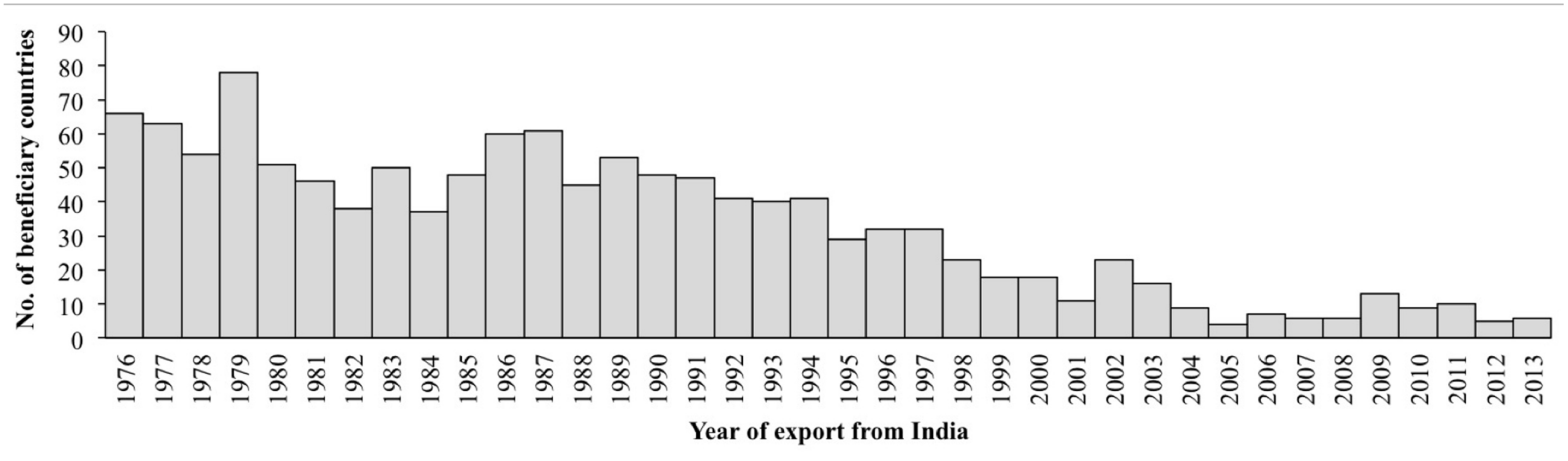
Year-wise profile of number of beneficiary countries that received germplasm from India.

A total of 91,403 germplasm accessions were supplied by India during the time span of 38 years. The temporal trend in germplasm export is depicted in Fig 2 along with the export requests/indents received by India. The number of beneficiary countries of Indian accessions is depicted in Fig 3. The pre-1980s data revealed bulk export of germplasm by India, in line with the international policy of free exchange of germplasm that existed during that period. The number of export requests received was also simultaneously high (Fig 2). The IRRI, USDA genebanks and AVRDC were the major beneficiaries of Indian-origin germplasm during this period. Amongst individual countries, Australia, erstwhile USSR and UK were the major importers of Indian germplasm and more than 60 other countries (maximally 78 during 1979) were recipients every year (Fig 3). Subsequently, i.e. from 1980–2000, there was a lesser, but still consistent, flow of germplasm from India. This period covered the various phases of the global CBD policy regime, including its transition phase. During this period, every year around 20 to 40 countries were the beneficiaries of Indian germplasm. The Biological Diversity Act (BDA), India’s own *sui generis* system for CBD compliance, was enacted in 2002 and its rules were framed in 2004, which coincided with significantly low export figures that continued for few years (Fig 2). There was an apparent reduction in the number of recipient countries as well (Fig 3). Subsequently the export scenario improved, though erratically, from 2008 onwards. The data on export requests/indents revealed that after 1992, a sharp decline was observed in requests/indents received by India (Fig 2). However, almost all the indents received, as depicted in the figure, were given positive supply response to the extent of 90–100%. The general reduction in export, wherever cited, coincides with a simultaneous low number of requests/indents received from other countries by India (Fig 2).

Rice and wheat were the major crops that were exported consistently from India. In total, Indian wheat germplasm has been supplied to 61 countries and rice germplasm has been supplied to 50 countries, which highlights the significant contribution of Indian-origin germplasm to global food security. Out of the total exported germplasm i.e. 91,403 accessions, CG genebanks, USDA genebanks and AVRDC, Taiwan, received a total of 23,904 (26%) Indian-origin accessions, of which most germplasm belonged to cereals, the main staple food crop group that contributes **>**60% of the world’s food energy intake [5], followed by vegetable crops which contribute significantly to nutritional security. These 23,904 exported accessions are in fact, India’s direct contribution to the 62,920 Indian-origin accessions held by the CG genebanks, as depicted in Genesys. Additionally, India has also facilitated the export of 150,688 accessions from ICRISAT, India, mostly to its sub-centres, which also included an undeclared number of Indian-origin accessions.

All these accessions received by the CG centres were freely available throughout the germplasm flow chain and thereby, are currently held by multiple global institutes to the tune of 106,007 accessions, as reported by Genesys. Here, it is important to mention that developing countries are globally acknowledged to be stronger donors of germplasm material than developed countries [22]. Wherever, India had recorded a declining trend in germplasm export, as in the late 1990s, it was not a ‘stand-alone’ example for this stringency. A downward trend for accessibility and sharing of germplasm was observed globally, including CG genebanks, since 1998 [22] due to the transformation of international agreements particularly with regard to ABS related issues [17, 25]. However, it did not nullify the germplasm flow to any critical extent. Hence, this decline in supply is not to be seen as a negative impact, but is rather the reflection of the affirmed balance that had developed within the realm of CBD and under obligations of the national legislations, wherein every country had already developed or was in the process of developing *sui generis* mechanisms to ensure a ‘fair’ flow and use of germplasm in accordance with their national IPR regime. Such a policy climate influenced the general demand-supply process of germplasm exchange, as evident from the lower number of export indents, and India *per se* has not withheld itself from the supply requirements during any policy period. It should also be noted that once the CG genebanks established themselves as the major custodians of plant biodiversity, the global trend of germplasm supply shifted to the CG domain [17, 18, 26], as against the nations having Vavilovian centres of crop origin [27], which were approached by the users in a limited manner.

This analysis does not attempt, in any manner, to undermine the significance of the exotic germplasm material received by India during the course of time, irrespective of the source. India is a recipient of a large amount of germplasm over the period of time from multiple donors including CG genebanks and other national genebanks [22]. The national agricultural research system in India has received synergistic support from such introductions, which has contributed significantly to Indian food security. However, the rationale behind the concept of ‘providers’ and ‘users’ should be redefined on the basis of a wider time frame that encompasses multiple policy regimes. Accordingly, in pre- and post-CBD era, India has never been less of a ‘provider’ as evident by the germplasm flow over the past four decades, which is unrestrictedly available to the global community through the CG genebanks under SMTA and other national genebanks under bi- and multi-lateral agreements. Still, the truth stands out that the protective mechanism offered by CBD was definitely a boon to countries like India which is a goldmine of genetic diversity in many food and fodder crops. It also provided a legal platform for scrutinizing the germplasm flow and siphoning the due benefits to authorized beneficiaries within the country through the National Biodiversity Fund created under BDA, 2002.

### Indian-origin germplasm and current ABS regime

Though the debate on the status of collections conserved as *ex situ* collections outside their country of origin has been prevalent since the CBD enforcement, its relevance cannot be undermined even today, after the CBD-NP enforcement in 2014 and operationalization of the MLS in 2004. During the CBD negotiations, PGR acquired prior to its entry into force were excluded from its ambit. The ITPGRFA was implemented to resolve the issue by culling out the 64 listed PGRFA from the purview of the CBD and developing a MLS for generating a common gene pool for use by all the member nations.

Although the objectives of CBD and the ITPGRFA are common in terms of promoting conservation and sustainable use of the genetic diversity and the equitable sharing of benefits derived from its use, the ABS mechanisms are quite different. Whereas the ITPGRFA achieves ABS by international pooling and sharing of PGRFA, the CBD-NP allows each country to carefully control access to its sovereign genetic resources, subject to individually tailored benefit-sharing agreements (monetary and non-monetary). The ITPGRFA, through Article 13.2, provides for benefit sharing through mechanisms such as exchange of information, access to and transfer of technology, capacity building, and sharing of monetary and other benefits of commercialization. The monetary benefits under the ITPGRFA are accessible through two funding mechanisms—the Benefit Sharing Fund (BSF) of the ITPGRFA and the Global Crop Diversity Trust (GCDT). The guidelines of the funding strategy for the implementation of the ITPGRFA have a broad range of criteria for selection. These include project relevance, feasibility, effectiveness and efficiency in terms of budget, immediate beneficiaries and contribution to economic development, team composition, capacity and collaboration, planning and monitoring, sustainability and geographic extension. One of the critical considerations is crop relevance, wherein the relevance of a crop to the quality and diversity of the human diet or animal feed, conservation and utilization of the crop’s gene pools, its centres of diversity and threat status are considered. The selection criteria do not give any weightage to a country’s contribution to the global food platter in the past. Inclusion of such a measure would be a great incentive for sharing of the germplasm in the MLS, one of the major objectives of the ITPGRFA. Commensurate benefit sharing funding with share of germplasm to the global pool would act as positive feedback mechanism for increasing germplasm in the MLS. This imbalance can be viewed from the fact that amongst the 30 projects funded under the BSF only two have been awarded to India. Benefit sharing in terms of technology acquisition, capacity building and access to advanced seeds/propagules needs to be linked with contribution of germplasm by member countries (directly or indirectly) into the MLS.

The interface between the ITPGRFA and NP is complex and decision tools to support such mechanisms are still in the process of development in most countries including India. Article 4.1 of the NP concerning its ‘relationship to other agreements’ provides that it shall not affect the rights and obligations relating to other international agreements, stating that the protocol will be ‘implemented in a mutually supportive manner’ with other international instruments. In the same provision, it is clarified that it does not create a hierarchy between the NP and other international instruments making all potential Vienna Convention-interpretation arguments inoperable. Article 4.2 leaves space for bilateral or international agreements regulating ABS under the precondition that these are CBD and NP-compliant. Considering that genetic resources are very often shared by a number of different countries and communities, Article 10 of the NP refers to a global multilateral benefit sharing mechanism. Article 10 provides, thus, for a similar approach to that of the ITPGRFA Multilateral System. As far as PGRFA are concerned, countries like India need to visit these provisions with utmost care while harmonizing the national laws with CBD-NP and WTO-TRIPs, especially with respect to benefit sharing.

## Conclusions

The PGRFA sustain global agriculture through cohesive efforts involving almost all countries which are bound together by multiple legal instruments on the international platform, especially in the context of germplasm exchange and benefit sharing. The CG genebanks have the most predominant role as germplasm suppliers and individual countries, that are otherwise the seat of genetic diversity, tend to stay in the background. Nevertheless, the contribution made by individual countries to CG genebanks cannot be undermined. Though, countries like India, which fall in the latter category, have all the while been responding positively to the supply requests for plant germplasm and to multiple developments at the global policy front, there is an apparent cautiousness with which each step is being put forward. The obligation under the ITPGRFA demands unrestricted sharing of indigenous germplasm belonging to Annex 1 crops in the global domain, under the SMTA mechanism. One of the major concerns is whether, with the foregoing of the case to case basis transaction mechanism, as done under the post-CBD era, India is going to lose direct control over utilization and benefit acquirement of its indigenous germplasm accessions. There is similar concern over the utilization of the germplasm provided by India in the past, which is currently held by multiple international organizations including CG centres under the purview of their respective legal clauses that are outside India’s legal ambit. The norms for sharing of such germplasm accessions are currently not uniform across institutions as has been cited in the case of several mega hybrid consortium projects involving multinational companies, where the CG genebanks are sharing germplasm with the private sector on payment basis [17]. The concept of minimizing cost of germplasm transactions by centralizing the conservation strategy also requires a more objective analysis since accumulation of resources at a single point invites more issues than mere cost benefits that are often cited. A frequently raised concern is how much cost (inclusive of conservation) should an accession be tagged with? It should be as minimal as possible according to SMTA provision. But while trying to minimize this cost through a centralized distribution mechanism, we cannot afford to overlook the ‘base price’ incurred at the actual source site of the germplasm wherein it had evolved over the years and to which multiple factors specific to that ecology would have positively contributed. These concerns may sound minuscule when addressed amongst the numerous issues pertaining to the germplasm access and benefit sharing. However, they have large implications for developing countries like India where ~70% of the population is dependent on agriculture for their daily sustenance and livelihood. These people have directly or indirectly, intentionally or unintentionally, contributed and invested to the conservation and sustainable utilization of valuable PGRFA, which is now available for the benefit of the global agricultural research community.

Generally, laws are formulated and enforced, without individual’s choice, for certain ethical features that are deemed fundamental to societal living and human co-existence [28]. In the same spirit CBD laws were formulated for fair exchange, sharing of benefits and use of genetic resources based on the principle of justice. Benefit sharing laws need to be enforced in such a way that the issue of mistrust is removed between ‘provider’ and ‘user’ and it motivates the parties to exchange the germplasm with a mechanism of fair and equitable sharing of benefits. The fundamental principle of giving a large stake in the benefits that flow from natural resources, to their custodians, in return for better chances to preserve our planet’s biodiversity for the benefit of human beings everywhere, present and future, should be adhered to in letter and spirit. In the context of increasing criticism from developing countries regarding the exploitation of their biological resources, it is much more likely that access for use will be granted freely, if developing countries’ concerns are satisfactorily addressed through ABS agreements that are tangible and show the visible impact. Considering the fact that out of the world’s 6.7 billion people, over one billion residing in gene-rich but least developed or developing nations are chronically undernourished and about 2 billion lack access to essential medicines, therefore, sharing of benefits arising from the use of PGR is not only an extremely pressing need but also part of the natural common justice.

## Supporting Information

S1 Table Indian germplasm deposited and conserved in SGSV, Svalbard, Norway. The genebanks are listed by ‘No. of accessions of Indian origin’ in descending order. The table lists the 24 national and international genebanks which have deposited Indian-origin germplasm in SGSV, Norway. The total accessions submitted by each institute till 31 August, 2014 and the number of Indian-origin accessions within the submitted germplasm are depicted.
(DOCX)
