# Correction: Indian Plant Germplasm on the Global Platter: An Analysis

**DOI:** 10.1371/journal.pone.0132087

**Published:** 2015-06-26

**Authors:** Sherry R. Jacob, Vandana Tyagi, Anuradha Agrawal, Shyamal K. Chakrabarty, Rishi K. Tyagi

There is an error in the fifth sentence of the Abstract. It should read: A total of 2,802,770 accessions are being conserved world-wide by 446 organizations represented in Genesys.

There are errors in Table 1. Please see the corrected Table 1 here.

**Table 1:** Germplasm of Indian-origin conserved in CGIAR genebanks. The table lists the number of accessions of Indian-origin, conserved in the 11 CG genebanks. The genebanks are listed by ‘No. of accessions of Indian origin’ in descending order.

S. No.	CGIAR Genebank	Total no. of accessions	Accessions of Indian origin	
			No.	%
1	International Crop Research Institute for the Semi-Arid Tropics, India	119,524	37,470	31.35
2	International Rice Research Institute, The Philippines	131,862	17,824	13.62
3	International Centre for Agricultural Research in Dry Areas, Syria	147,118	3,747	2.55
4	International Institute of Tropical Agriculture, Nigeria	27,232	2,276	8.36
5	International Livestock Research Institute, Ethiopia	20,229	501	2.48
6	Centro Internacional de Agricultura Tropical, Colombia	64,721	422	0.65
7	Centro Internacional de Mejoramiento de Maíz y Trigo, Mexico	164,320	318	0.19
8	West African Rice Development Association, Ivory Coast	26,098	299	1.15
9	*Musa* International Transit Centre, Bioversity International, Belgium	1,529	54	3.53
10	Centro Internacional de la Papa, Peru	16,061	9	0.06
11	Information and Communication Division, International Center for Research in Agroforestry, Kenya	2,005	0	0
	**Total**	**720,699**	**62,920**	**8.73**
