# Supplementary material for: Indian Plant Germplasm on the Global Platter: An Analysis
Source: PLoS One. 2015 May 14;10(5):e0126634. doi: 10.1371/journal.pone.0126634 (PMC4431847; doi:10.1371/journal.pone.0126634)
Supplement: S1 Table — The genebanks are listed by ‘No. of accessions of Indian origin’ in descending order. The table lists the 24 national and international genebanks which have deposited Indian-origin germplasm in SGSV, Norway. The total accessions submitted by each institute till 31 August, 2014 and the number of Indian-origin accessions within the submitted germplasm are depicted. (DOCX) [file pone.0126634.s001.docx]

**S1 Table.** Details of 24 national and international genebanks which have deposited Indian origin germplasm in SGSV, Svalbard, Norway. The genebanks are listed by ‘No. of accessions of Indian origin’ in descending order.

| **S. No.** | **Name of the Institute** | **No. of total accessions conserved** | **No. of accessions of Indian origin** | **No. of species of Indian origin** |
| --- | --- | --- | --- | --- |
| 1 | International Crop Research Institute for the Semi-Arid Tropics, India (IND002) | 104,000 | 33,424 | 12 |
| 2 | International Rice Research Institute, the Philippines (PHL001) | 116,668 | 16,220 | 18 (including crosses) |
| 3 | National Plant Germplasm System, USA (USA996) | 69,307 | 4,934 | 97 |
| 4 | The World Vegetable Center – AVRDC, Taiwan (TWN001) | 12,769 | 4,015 | 22 |
| 5 | International Centre for Agricultural Research in Dry Areas – ICARDA, Syria (SYR002) | 116,484 | 3,567 | 15 |
| 6 | International Institute of Tropical Agriculture, Nigeria (NGA057) | 18,813 | 2,353 | 5 |
| 7 | Centre for Genetic Resources, the Netherlands (NLD037) | 18,642 | 489 | 17 |
| 8 | Australian Grains Genebank, Australia (AUS165) | 7,486 | 379 | 9 |
| 9 | Centro Internacional de Agricultura Tropical –CIAT, Colombia (COL003) | 47,898 | 350 | 9 |
| 10 | Canadian Genetic Resources Program, Saskatoon Research Centre, Canada (CAN004) | 25,868 | 272 | 13 |
| 11 | Leibniz Institute of Plant Genetics and Crop Plant Research – IPK, Germany (DEU146) | 36,534 | 189 | 39 |
| 12 | Uzbek Research Institute of Plant Industry (UZB006) | 2,038 | 176 | 5 |
| 13 | Africa Rice Center – WARDA, Benin (CIV039) | 12,439 | 124 | 1 |
| 14 | N.I. Vavilov All-Russian Scientific Research Institute of Plant Industry, Russia (RUS001) | 5,278 | 71 | 11 |
| 15 | National Agricultural Research Organization, Uganda (UGA031) | 777 | 49 | 1 |
| 16 | Station Fédérale de Recherches en Production Végétale de Changins , Switzerland (CHE001) | 9,665 | 32 | 1 |
| 17 | International Livestock Research Institute, Ethiopia (ETH013) | 5,335 | 31 | 8 |
| 18 | National Bureau of Plant Genetic Resources, India (IND001) | 25 | 25 | 1 |
| 19 | Nordic Genetic Resources Center, Sweden (SWE054) | 15,265 | 16 | 3 |
| 20 | Seed Savers Exchange, USA (USA974) | 2,408 | 10 | 4 |
| 21 | The Brazilian Agricultural Research Corporation, Brazil (BRA008) | 1,319 | 5 | 1 |
| 22 | Institute of Plant Production n.a. V.Y.Yurjev of UAAS, Ukraine (UKR001) | 2,782 | 3 | 2 |
| 23 | World Agroforestry Centre, Kenya (KEN023) | 777 | 3 | 1 |
| 24 | Biotechnology, Plant Genetic Resources and Plant Protection Division, Myanmar (MMR 003) | 718 | 2 | 1 |
|  | **Total** | **633,295** | **66,739** |  |
